# Accelerometry and salivary cortisol response during Air Force Special Tactics Officer selection

**DOI:** 10.1186/2046-7648-2-28

**Published:** 2013-10-01

**Authors:** John S Cuddy, Andrew R Reinert, Walter S Hailes, Dustin R Slivka, Brent C Ruby

**Affiliations:** 1 Department of HHP McGill Hall, The University of Montana, Center for Work Physiology and Exercise Metabolism, 32 Campus Drive, Missoula, MT 59812, USA; 2 720th Special Tactics Group, 223 Cody Avenue, Hurlburt Field, FL 32544, USA; 3 School of Health Physical Education and Recreation, University of Nebraska at Omaha, HPER 207R, 6001 Dodge Street, Omaha, NE 68182, USA

**Keywords:** Activity monitoring, Energy expenditure, Military, Special forces

## Abstract

**Background:**

Special Tactics Officer (STO) selection is conducted to select officers to enter the combat controller training pipeline. The aims were to determine physical activity patterns, estimate energy expenditure, and identify whether return and/or unsuccessful candidates demonstrated differences in cortisol responses compared to non-selected and/or first-time attendees.

**Methods:**

Participants completed the STO selection, consisting of 5 days of physical and mental challenges. Participants were equipped with ActiCals®, and saliva samples were collected throughout the STO selection.

**Results:**

Average activity counts were 684 ± 200 counts∙min^−1^, with no group differences. Estimated energy expenditure was 4,105 ± 451 kcal∙day^−1^. Cortisol was elevated following extended physical training but returned to baseline during rest. Return candidates had significantly lower cortisol responses compared to first-timers, 0.43 ± 0.06 μg∙dl^−1^ versus 0.76 ± 0.18 μg∙dl^−1^, respectively, *p* < 0.05.

**Conclusions:**

An individual's salivary cortisol response to the stresses incurred during the STO selection has the potential to be incorporated into the entire picture of a candidate's performance and ability to handle stress.

## Background

United States Air Force Special Tactics Officer (STO) selection is conducted biannually in an effort to select officers who possess the necessary leadership qualities to enter the combat controller training pipeline. Combat controllers are elite Special Operations soldiers who possess a high level of fitness, specialized combat skills, sky diving, parachuting (static line and free fall), scuba training, and various weapon qualifications. Combat controllers specialize in airfield seizure and control, call for fire on targets (dropping bombs, guided weapons, artillery), controlling close air support, and target acquisition. Combat controllers work in close cooperation with other Special Operations Forces (SOF), including Army SOF and Navy Sea, Land, and Air teams. As a SOF ground combatant force, they maintain the same or higher physical attributes of strength, stamina, and endurance as other elite SOF. Identifying selection measures of SOF combatants is complex, but physical prowess, motivation, and spatial ability have been recognized as key factors [1]. Additionally, officers require strong leadership skills, and the ability to think clearly during stressful, ambiguous situations.

While many stressors involved in the STO selection are physical (ruck marching, pool sessions, limited caloric intake, etc.), a considerable component of the selection process is related to the qualities of mental resiliency that candidates exhibit during confusing and stressful circumstances. The difficulty of the course is compounded further by consistent sleep deprivation, magnifying the psychological/cognitive stress upon candidates. Candidates attempt to solve problems that may not have solutions and work together to achieve specific outcomes. Collectively, the physical and mental components of the STO selection combine to increase the overall stress load on candidates during the week-long selection process.

When exposed to acute physical and/or mental stresses, a cascade of hormones, including cortisol, prepares a person for physical movement and/or protection. Cortisol is a glucocorticoid that assists in partial regulation of carbohydrate, fat, and protein metabolism, and can be used as an acute and chronic indicator of stress [2]. While diurnal fluctuation in cortisol is normal [3], an acute increase in cortisol can be caused by both psychological [4-7] and physiological [8-10] stimuli. In addition, cortisol responds to changes in training status and performance during the course of a sport season [9]. In several studies observing stress responses to simulated prisoner of war camps, Morgan et al. found acute increases in cortisol in response to stressors placed upon participants [11-14]. Cortisol increased in response to survival training stress [13], and high cortisol was associated with greater subjective distress [11], greater dissociation (transient sense that the world or the self is ‘unreal’) during [12] and following stress [14], and reduced military performance [12]. In elite golfers, cortisol levels increased prior to competition, but there was no relationship between cortisol levels and performance [15]. In contrast, during weightlifting competition, an acute influx of cortisol prior to competition was beneficial for improving performance [16]. It is difficult to ascertain whether increased cortisol prior to competition always improves performance [17-19], but the heightened state of being, ‘fight or flight,’ prepares athletes for competition. Thus, elevated cortisol levels may be beneficial or detrimental to physical and/or mental performance during stressful situations depending on the task at hand. It is likely that when tasks involve gross motor skills (i.e., weightlifters), an enhanced stress response is beneficial, whereas it may not be as helpful when fine motor skills are necessitated. During the ‘fight or flight’ response, cortisol directly prepares the body for movement by mobilizing glucose into the bloodstream, increasing brain's use of glucose, altering immune response, and suppressing the digestive system.

Although past research has established the typical energy expenditure [20] for military operations, descriptions of daily activity patterns have been limited [21-23]. Activity monitors can estimate energy expenditure and quantify physical activity patterns, making them a practical, simple, non-invasive research tool. The use of monitors for tracking activity has been used in diverse subject populations [21-27]. A primary advantage of using activity monitoring is the ability to classify activity into different metabolic intensities, revealing how hard participants work. Analyzing alterations of cortisol alongside activity data may provide indicators of candidates' resiliency to stressful situations.

The high physical and psychological strain associated with SOF selection provides an attractive model to quantify how the human stress response may be associated with successful task completion. Therefore, the aims of the current study were to determine physical activity patterns, estimate energy expenditure, and identify whether return and/or successful SOF candidates demonstrated differences in cortisol responses compared to non-selected and/or first-time attendees.

## Methods

The research study was approved by the Institutional Review Board at The University of Montana and Air Force Research Laboratories, Wright Site Institutional Review Board. Prior to beginning the study, researchers briefed participants on the requirements for being a subject and made clear that participation in this study would in no way affect the outcome of the STO selection. Participation in the study was voluntary, and subjects provided written informed consent. Data collection took place at Hurlburt Field, FL, USA.

### Subjects

Subjects were candidates (*n* = 11, mass 76 ± 6 kg, height 177 ± 9 cm, and age 26 ± 3 years) striving to become Special Tactics Officers. Selected candidates (*N* = 4) were those picked by the cadre to enter the combat control pipeline as officers; they were chosen at the end of the selection course. Repeat (*N* = 3) and first-time attendees (*N* = 8) were designated by asking whether they had previously attended the STO selection course. Of the four successful candidates, three repeat candidates were selected, and one first-time candidate was selected. One subject was unable to pass the initial PT tests and was eliminated the first night of the study. Two subjects voluntarily eliminated themselves from the selection process on day 3 of the study. Two candidates were unable to complete the final event due to injury. One first-time non-selected candidate had to be dropped from cortisol analysis due to sample contamination at one time point. Activity data represents the six participants who completed the entire selection process (five candidates were first-time attendees, while one was a repeat attendee), and salivary cortisol represents nine participants through day 3 and seven participants through day 5.

### Experimental design

Candidates participated in a wide range of different activities during the 5-day selection process, including: running, swimming, calisthenics, ruck marching, water skill sessions, leadership reaction courses, and ‘Monster Mash’ (a several-hour mission that included swimming, land navigation, ruck marching, load carrying, and skill tests). A specific time frame of events is not available for public distribution due to the need for the course to remain unpredictable and ambiguous for future candidates. During the STO selection course, subjects participated in the following: (1) pre and post body mass, (2) measurement of activity patterns by wearing ActiCal® activity monitors on the wrist, and (3) saliva samples were collected at 11 time points to assess changes in cortisol.

### Body mass and height

Subjects' body mass was measured using a digital scale (Detecto, Model-758C, Webb City, MO, USA) at the beginning and end of the study. Height was measured using a stadiometer (Narragansett Machine Co, Providence, RI, USA). For all measurements, subjects wore socks and training shorts.

### Physical activity patterns and total energy expenditure

Activity was measured by placement of a small ActiCal® activity monitor (MiniMitter, Bend, OR, USA) with an adjustable hospital band on the non-dominant wrist with the blue arrow pointing toward the elbow [28]. The wrist location has been previously used with accelerometry measurement during military research [21-23]. The monitor was worn continually for the 5-day study. Daily energy expenditure was calculated indirectly using ActiCal® 2.0 software (MiniMitter, Bend, OR), and task-specific energy expenditure was estimated using previously established algorithms [28]. Energy expenditure for the run portion of the study was calculated using the American College of Sports Medicine running metabolic equation [29], and energy expenditure for the swim portion was calculated using the *Compendium of Physical Activities*[30]. Activity intensity was classified based upon the following cut points: sedentary and light (0 to 144 kcal∙h^−1^), moderate (145 to 386 kcal∙h^−1^), and vigorous (387+ kcal∙h^−1^) [28]. To discriminate between energy expenditure in the sedentary and light categories, activity monitor cut points were used: sedentary (0 to 50 counts∙min^−1^) and light (50 to 600 counts∙min^−1^).

### Salivary cortisol

**Figure 1:**
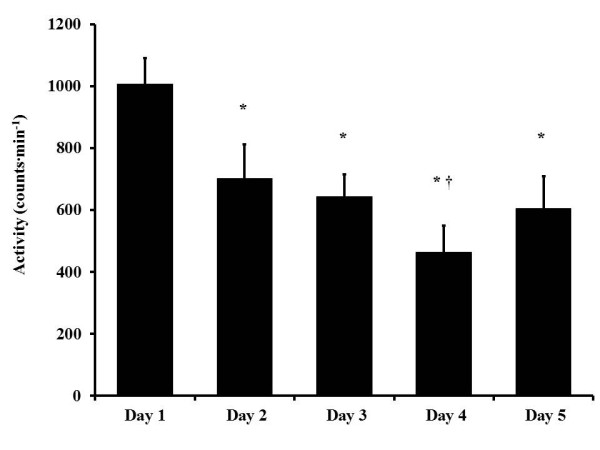
**Activity and salivary cortisol during the STO selection.** Single asterisk (*) *p* < 0.05 first-time candidate cortisol compared to repeat candidate cortisol over 5 days of the STO selection, main effect of group.

Saliva was collected (approximately 3 ml) using passive drool and frozen at −30°C. Samples were taken following a minimum of 15 min without food or fluid intake. Prior sleep before sampling varied day to day, but participants had similar sleep opportunities and conditions. Salivary cortisol was measured using a competitive immunoassay on a micro-plate reader (Model 680 XR, Bio-Rad, Hercules, CA, USA) at 450 nm in accordance with the manufacturer's protocol (Salimetrics, State College, PA, USA). Salivary cortisol was collected at 11 different time points throughout the study (two times per day: upon waking (times varied) and at approximately 1,100, see Figure 1). Though sample times varied day to day, all participants provided samples at the same time, every time. A potential limitation of the study is that sample times were not controlled each day, though the effect of the course (altered wake-rest cycles) on normal diurnal variation may dilute this limitation.

### Data analysis

All descriptive data were expressed as means ± standard deviation. A dependent *t* test was used to determine differences in body mass from day 1 to day 5. Activity counts and energy expenditure were compared using a one-way analysis of variance across time. To compare cortisol, data across time and between repeat versus first-time candidates and selected versus non-selected were analyzed using a mixed design analysis of variance (group × time) with repeated measures for time. The Bonferroni correction was used for the adjustment of multiple comparisons. Statistical significance was established using an alpha level of *p* < 0.05.

## Results

### Body mass

There was no significant change in body mass (*N* = 7) from day 1 to day 5 (77.8 ± 3.1 kg and 77.6 ± 3.2 kg for pre and post, respectively).

### Activity data

**Figure 2:**
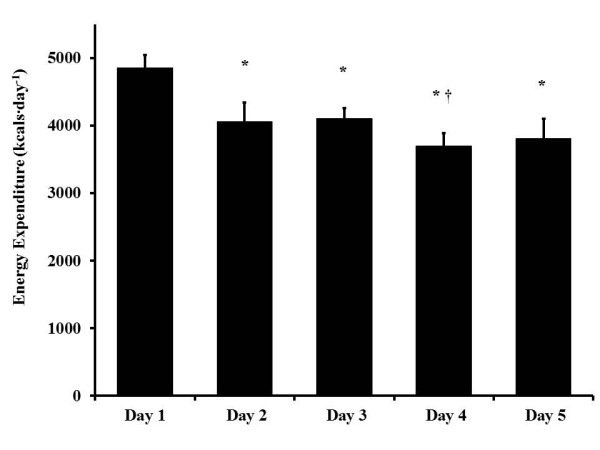
**Activity counts during the STO selection (*n*** **= 6).** Values are expressed as mean ± SD. Single asterisk (*) *p* < 0.05 compared to day 1. Dagger (†) *p* < 0.05 compared to day 3.

The average daily activity counts for the six subjects who completed the entire selection process were 684 ± 200 counts∙min^−1^. Activity during days 2 to 5 was significantly less than on day 1, *p* < 0.05 (Figure 2). Day 4 was lower than day 3 (*p* < 0.05). Time spent in different intensities and task-specific activity counts are detailed in Tables 1 and 2. Too few participants completed the ‘Monster Mash’ on day 5 (*n* = 6, only one candidate who was a repeat) to statistically compare between groups for activity and energy expenditure.

**Table 1:** Activity intensity profile of the STO selection

**Intensity**	**Number**	**Duration (h∙day**^**−1**^**)**	**Estimated energy expenditure (kcal∙day**^**−1**^**)**	**Activity (counts∙min**^**−1**^**)**	**Percent of total time**
Sedentary	6	8.7 ± 2.0	652 ± 142	16 ± 4	36 ± 8
Light	6	9.3 ± 1.4	1,483 ± 214	304 ± 14	39 ± 6
Moderate	6	4.7 ± 0.5	1,362 ± 158	1,493 ± 79	20 ± 2
Vigorous	6	1.2 ± 0.5	608 ± 326	5,580 ± 1,122	5 ± 2

Values are expressed as mean ± SD.

**Table 2:** Task specific activity counts, duration, and energy expenditures

**Activity data**	**Number**	**Sessions**	**Activity (counts∙min**^**−1**^**)**	**Duration (min∙session**^**−1**^**)**	**Estimated energy expenditure (kcal∙session**^**−1**^**)**
PT test calisthenics	10	1	2,145 ± 232	31 ± 0	123 ± 15
PT test run	10	1	13,127 ± 2,736	22 ± 1	392 ± 30^a^
PT test swim	10	1	5,082 ± 1,256	29 ± 3	384 ± 38^b^
Ruck marching	9	3	1,940 ± 350	193 ± 36	769 ± 119
Pool sessions	7	2	1,389 ± 882	183 ± 7	584 ± 84
LRC	9	2	565 ± 153	246 ± 37	600 ± 116
Night LRC	7	1	790 ± 160	369 ± 0	1,062 ± 88
Monster Mash	6	1	1,789 ± 627	293 ± 0	1,155 ± 186

Values are expressed as mean ± SD. LRC, Leadership Reaction Course; PT, physical training. ^a^Kilocalories derived from [29]. ^b^Kilocalories derived from [30].

### Energy expenditure

**Figure 3:**
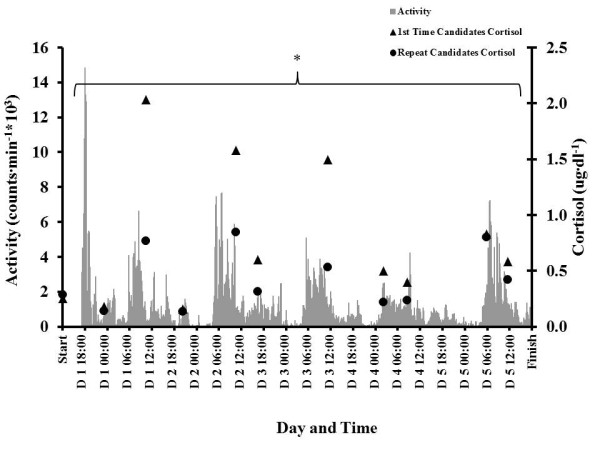
**Estimated daily energy expenditure during the STO selection (*n*** **= 6).** Values are expressed as mean ± SD. Single asterisk (*) *p* <0.05 compared to day 1. Dagger (†) *p* < 0.05 compared to day 3.

The average daily estimated energy expenditure for the six subjects who completed the entire selection process was 4,105 ± 451 kcal∙day^−1^. Energy expenditure during days 2 to 5 was significantly less than day 1, (*p* < 0.05, Figure 3). Day 4 was significantly lower than day 3 (*p* < 0.05). Energy expenditure associated with specific tasks is detailed in Table 2.

### Salivary cortisol and activity

Salivary cortisol (*N* = 7) was elevated (*p* < 0.05) compared to baseline (day 1, 13:00) following 4- to 5-h physical training sessions (time points 3, 5, and 7) but recovered to similar levels as baseline when candidates were provided times of reduced activity (Figure 1).

### Group differences

**Figure 4:**
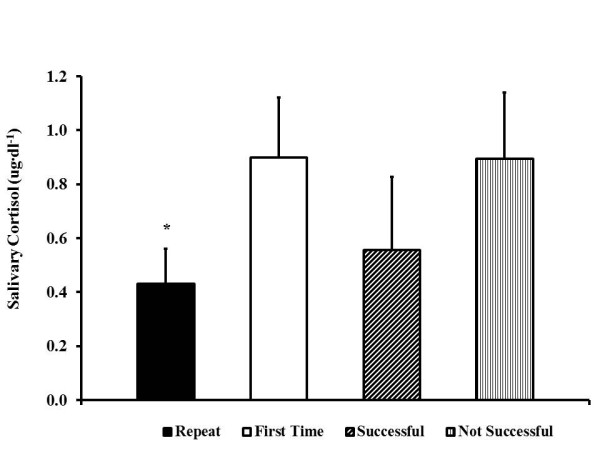
**Mean salivary cortisol during the first 3 days of the STO selection.** For return (*n* = 3), first-time (*n* = 6), successful (*n* = 4), and not successful (*n* = 5) candidates. Values are expressed as mean ± SD. Single asterisk (*) *p* < 0.05 compared to First Time.

First-time candidates (*n* = 6) had higher cortisol than return candidates (*n* = 3) (0.90 ± 0.22 μg∙dl^−1^ versus 0.43 ± 0.13 μg∙dl^−1^, respectively, *p* < 0.05) during the first 3 days (time points 1 to 7) (Figure 4). Two of the six first-time candidates withdrew after day 3. When the remaining four first-time candidates were compared to the three return candidates for the entire 5-day period of the selection process, there was still a lower cortisol response for those who had previously attended, 0.43 ± 0.06 μg∙dl^−1^, compared to 0.76 ± 0.18 μg∙dl^−1^ for first-timers, *p* < 0.05. There was a trend toward a difference between selected (*N* = 4) and non-selected candidates (*N* = 5) for cortisol, (0.56 ± 0.27 μg∙dl^−1^ and 0.89 ± 0.25 μg∙dl^−1^, respectively, *p* = 0.09) (Figure 4).

## Discussion

Special Tactics Officer selection is physically and mentally demanding, with estimated energy expenditures of 4,105 ± 451 kcal∙day^−1^. Candidates appeared to do an adequate job maintaining fluid and energy balance, demonstrated by no change in body mass over the 5-day selection process. The activity data contribute to a growing body of military research using accelerometry as a way to assess activity patterns during sustained military operations [21-23]. Activity patterns are important because they can show work/rest cycles with high resolution (minute by minute), as well as intensity of activity, over an extended period of time (up to 44 days for the ActiCal®). The primary finding in this study was that return candidates had a reduced cortisol response compared to first-time participants despite similarities in the estimated total energy expenditure (TEE) and a trend for higher activity for return candidates.

### Energy expenditure

The reduction in energy expenditure over the course of the study suggests that the STO selection became progressively less physically demanding. The estimated energy expenditure in this study (4,105 ± 451 kcal∙day^−1^) was similar to other military scenarios, with the average 4,610 ± 650 kcal∙day^−1^[20]. However, these scenarios had an average time frame of 12.2 days, whereas the current study was 5 days [20]. Previous research completed during a 10-day Marine Officer Selection Course showed a mean daily energy expenditure of 5,378 kcal∙day^−1^ (66.4 kcal∙kg^−1^) [31] (calculated from doubly labeled water). In contrast, the present 5-day mean estimate for the STO selection TEE was 53 ± 6 kcal∙kg^−1^, 25% lower than the Marine Corps study. The ActiCal® does not account for load carriage (a substantial component of STO selection) when estimating energy expenditure, and since load carriage increases energy expenditure [32], this error may partially account for the discrepancy between studies. There were minimal, non-significant differences between groups for energy expenditure (5,139 ± 342 and 5,495 ± 136 kcal∙day^−1^ for the first-time and return candidates, respectively).

### Activity patterns

High levels of physical and mental stamina are necessary for completion of this course, and activity monitoring provides quantifiable data on the physical aspects of the STO selection. However, a limitation of the ActiCal® activity monitor is accurately discerning the intensity of activity when participants are carrying a load, which occurred at certain times during this study. The bulk of activity during the STO selection was light and moderate intensity, collectively making up approximately 59% of the daily time. Vigorous activity made up approximately 71 min∙day^−1^. The run, swim, and portions of ruck marching were the most intense physical activities during the 5-day selection process. Based on activity counts, each candidate averaged approximately 5 min similar to running and approximately 15 min similar to swimming during each ruck marching session (carrying an approximately 27-kg pack). Thus, portions of ruck marching were arduous even though the ActiCal® did not account for load carriage. Future research could determine ActiCal® activity counts and associated energy expenditure during higher intensity activities and loaded carrying, which would provide better resolution for the metabolic intensities of the STO selection.

### Salivary cortisol

During the 5 days of the STO selection, there were daily fluctuations in salivary cortisol (Figure 3), and these changes were dictated by the activity pattern prior to the collection point. The extended duration and high-intensity nature of the exercise prior to time points 3, 5, and 7 explain the heightened cortisol response [8,33,34]. During times of less physical demand, salivary cortisol recovered to baseline levels, indicating that candidates were well stressed from different tasks but recovered adequately. Though not statistically analyzed, of particular interest are time points 10 and 11, pre and post ‘Monster Mash.’ The activity data suggests ruck marching, pool sessions, and the Monster Mash are similar to one another (1,940, 1,389, and 1,789 counts∙min^−1^). However, the salivary cortisol response to tasks on days 1, 2, and 3 (the three highest cortisol data points) is considerably higher than the post ‘Monster Mash’ time point. Prior to the Monster Mash, candidates had their lowest activity period (298 ± 81 counts∙min^−1^) in the preceding 21 h, including approximately 7.5 h of sleep. This drop in physical activity provided time for physical recovery and, combined with possible adaptation to the mental stressors, might have been the reason for a reduced salivary cortisol response [5,10]. Additionally, it is possible that in just a few days, the first-time candidates have begun to cope with the uncertainties involved with the selection course and are more comfortable with the surroundings.

It is difficult to generalize the results because of the small number of subjects per group; results could have been due to individual differences. Accepting this limitation, there was a significant difference in the cortisol response between the candidates who had previously attended the STO selection compared to the candidates who were attending the STO selection for the first time. Average cortisol values were 43% lower for return candidates over 5 days, suggesting a reduced response to the stressors of the STO selection despite possible higher counts∙per minute for return candidates. It is difficult to ascertain a specific reason for reduced salivary cortisol levels in the returning candidates. Morgan et al. [14] suggest that SOF soldiers had a rapid release of neuropeptide-Y and norepinephrine and less difference in baseline/recovery cortisol, demonstrating greater tolerance to stressors than other soldiers. These soldiers were characterized by having greater ‘stress hardiness,’ and it is likely the return candidates in this study had been toughened by prior exposure to the STO selection. Additionally, since the candidates had a general expectation of the course (the STO selection is varied for every class), they might have had reduced anticipatory psychological stress [10] or physically prepared themselves more than first-time candidates. Thus, the physical tasks would not stress them as much, and their cortisol would return to baseline quicker than first-time candidates.

The current findings warrant further investigation. Three of the four candidates selected were return candidates; thus, it brings up the question whether reduced cortisol was a trait for return candidates or successful candidates. There was a trend for a reduced cortisol for those who were selected compared to those who were not (*p* = 0.09), and future research could expand this question with a larger study looking at the relationship of the cortisol response to the success of troops during stressful situations. Additionally, return candidates responded differently to stress and were more likely to be selected than first-time candidates (100% of return candidates were selected, while only 12.5% of first-time candidates were selected). Prior exposure could provide an advantage for those attending for the second time, or selected candidates have certain physiological responses to stress that set them apart from non-selected candidates.

## Conclusions

An estimate of the energy expenditure during the STO selection was 4,105 ± 451 kcal∙day^−1^, and cortisol increased and decreased in concert with activity patterns. Return candidates had a reduced cortisol response compared to first-time candidates, which suggests they handled stresses better. In order to apply the current data into practice, a larger study looking at the cortisol response and candidate success/failure is needed. If the results were similar to this study, commanders could use cortisol as an objective physiological marker of how well a candidate can handle stressful situations. However, given the difficulty in capturing cortisol in real-time, other real-time metrics should be explored alongside cortisol that might be easier to capture and provide objective data that could be used for decision making. In short, an individual's salivary cortisol response to the stresses incurred during the STO selection has the potential to be incorporated into the entire picture of a candidate's performance and potential to handle stress.

## Abbreviations

PT: Physical training; STO: Special Tactics Officer; SOF: Special Operations Forces; TEE: Total energy expenditure.

## Competing interests

The authors declare that they have no competing interests.

## Authors’ contribution

JC participated in study conception, data collection, statistical analysis, and drafting the manuscript. AR participated in study design and coordination/collection of data. WH participated in data collection, statistical analysis, and drafting the manuscript. DS participated in statistical analysis and drafting the manuscript. BR participated in study conception and drafting the manuscript. All authors read and approved the final manuscript.
